# The Moderating Effects of Sex and Age on the Association between Traumatic Brain Injury and Harmful Psychological Correlates among Adolescents

**DOI:** 10.1371/journal.pone.0108167

**Published:** 2014-09-30

**Authors:** Gabriela Ilie, Edward M. Adlaf, Robert E. Mann, Angela Boak, Hayley Hamilton, Mark Asbridge, Angela Colantonio, Nigel E. Turner, Jürgen Rehm, Michael D. Cusimano

**Affiliations:** 1 Division of Neurosurgery and Injury Prevention Research Office, St. Michael's Hospital, Toronto, Canada; 2 Social and Epidemiological Research, Centre for Addiction and Mental Health, Toronto, Canada; 3 Dalla Lana School of Public Health, University of Toronto, Toronto, Canada; 4 Department of Community Health and Epidemiology and Department of Emergency Medicine, Dalhousie University, Halifax, Canada; 5 Department of Occupational Science and Occupational Therapy, Toronto Rehabilitation Institute, Toronto, Canada; University of Florida, United States of America

## Abstract

**Background:**

Although it is well established that sex is a risk factor in acquiring a traumatic brain injury (TBI) among adolescents, it has not been established whether it also moderates the influence of other TBI psychological health correlates.

**Methods and Findings:**

Data were derived from a 2011 population-based cross-sectional school survey, which included 9,288 Ontario 7th–12th graders who completed anonymous self-administered questionnaires in classrooms. Response rate was 62%. Preliminary analyses found no evidence of nonresponse bias in the reporting of TBI. TBI was defined as a hit or blow to the head that resulted in a 5 minutes loss of consciousness or at least one overnight hospitalization due to symptoms associated with it. Reports of lifetime TBI were more common among males than females (23.1%, 95% CI: 20.5, 25.8 vs. 17.1%, 95% CI: 14.7, 19.8). Thirteen correlates were examined and included cigarette smoking, elevated psychological distress, suicide ideation, bully victimization (at school, as well as cyber bullying), bullying others, cannabis use, cannabis dependence and drug use problems, physical injuries, daily smoking, drinking alcohol, binge drinking, use of cannabis, and poor academic performance. Among the outcomes examined, sex moderated the relationship between lifetime TBI and cigarette smoking. In addition, sex and age jointly moderated the relationship between lifetime TBI and daily smoking, alcohol use and physical injuries. Late adolescent males who reported lifetime TBI, relative to females, displayed elevated daily smoking and injuries, whereas their females counterparts displayed elevated past year drinking. Possible bias related to self-report procedures and the preclusion of causal inferences due to the cross-sectional nature of the data are limitations of this study.

**Conclusions:**

TBI differences in outcomes need to be assessed for potential moderating effects of sex and age. Results have important implications for more tailored injury prevention efforts.

## Introduction

Traumatic brain injury (TBI) is a significant source of adolescent impairment, disability and years of life lost, and consequently an appreciable burden to health care systems worldwide [1]–[3]. TBI creates significant clinical challenges for medical practitioners and for the injured patient's family and caregivers. Physical and psychological consequences associated with TBI include physical (e.g., lack of balance, visual or auditory problems), psychosocial/emotional (e.g., depression, anxiety, tension/irritability) and cognitive/communicative (e.g., attention and/or memory issues, social cognition) deficits [1]–[2]. A 2009/2010 Canadian population survey found that adolescents aged 12–19 had the second highest likelihood of a past-year bodily injury, and the second-highest likelihood of a past-year head injury (after those aged 65 and older) with many head injuries remaining unreported [4]. Preventable brain injuries remain a blind spot, particularly in North America where hits to the head continue to be perceived as an expected occurrence in sports [5]. Indeed the Centers for Disease Control and Prevention (CDCP) showed that sports related TBIs among adolescents increased by 60% between 2001 and 2009, with male adolescents accounting for 71% of TBI- related emergency department visits [6]. For this reason CDCP declared TBI among teens a major public health issue [7]. The prevalence of adolescent TBI is only now slowly being established. In Ontario it has been estimated that one in seven students aged 11–20 sustained at least one TBI in their lifetime, but not in the past 12 months, and one in 18 sustained a TBI in the past year [8]. In the US and Europe, TBIs among 15–19 year olds are the second highest category of combined rates of TBI-related Emergency Departments visits, hospitalizations and deaths [3], [9], [10]. The high estimated costs associated with TBI and TBI-related disability in Canada (over $7 billion), US (over $60 billion), and the European Union (over $97 billion) have increased the need to better understand its mechanisms and co-occurrence with other harmful health risk conditions [11]–[15].

Despite the rise of TBI among youth, little is currently known about the antecedents and consequences of teen TBI and even fewer studies have examined gender differences among them (usually due to lack of power for the analyses needed). Changes associated with TBI are complex and can include neuropsychiatric sequelae such as suicide, anxiety, depression, memory and learning problems, insomnia, mood and stress related issues, and misconduct behaviours, which affect a large number of injured individuals, with significant costs for their social re-integration [2], [8], [12], [16], [17]. Moreover, substance abuse plays a significant risk factor for homelessness and violence among homeless populations [18]. Additionally, most research has examined adult populations and much less data is available from studies with adolescents.

Sex differences in the prevalence of adult TBI are well established with studies indicating that men are more likely than women to have experienced a lifetime TBI [14]–[17], [19]–[20]. Although sex has been demonstrated to be a risk factor in acquiring a TBI, it has not been established whether the influence of TBI on health outcomes is altered by sex [19]. The investigation of sex differences in TBI among adolescents is limited, with such work often restricted to adult TBI, particularly male injuries [20]–[22]. It has been proposed that this male emphasis may be due to higher incidence of TBI among men, especially resulting from sport activities as well as a historical under appreciation for the impact of concussions among women [5], [22]–[23]. To date, no studies have reported on the moderating effects of sex on the prevalence of adolescent TBI, however, the adult literature suggests that both men and women experience long-term consequences from trauma [21]–[28]. Whether these consequences are of the same severity is largely unknown. Studies that have examined adult TBI outcome following a blunt force trauma to the head during childhood, found differences in TBI outcomes long-term. Specifically, these studies have shown that women experience greater deficits in emotional functioning, whereas men have impairments to memory and cognitive functioning [24]–[28]. Although research investigating sex differences in adolescent TBI have not yet been reported (perhaps due to insufficient sample sizes needed to address sex associations) the possibility of differential sex associations influencing TBI occurrence and outcome post TBI is an important consideration in adolescent TBI prevention, clinical management and rehabilitation.

The objective of this study was to assess whether either sex, or sex and age both moderate the association between TBI and various psychological health TBI associates – specifically, academic performance, mental health, substance use, bullying, suicide and physical injuries.

## Methods

The study was approved by the Research Ethics Committees of the Centre for Addiction and Mental Health, St. Michael's Hospital, participating Ontario Public and Catholic school boards, and York University, which administered the surveys. The study was conducted according to the principles expressed in the *Declaration of Helsinki*. All participants provided their own consent in addition to parental signed consent. Our analyses are based on three samples of 7th–12th graders: a full sample of 9,288, and two half samples of 4,685 and 4,230. All data were derived from the Centre for Addiction and Mental Health's (CAMH) 2011 cycle of the Ontario Student Drug Use and Health Survey (OSDUHS), a stratified (region by school level strata), two stage (school, class) double clustered probability sample repeated every odd-numbered year among Ontario students enrolled in grades 7 through 12 (age range 11–20) in publicly funded schools (including public, Catholic, English and French language schools), and representing some 93% of the comparably-aged Ontario adolescent population. The following schools and classes were excluded from sampling: (1) private and institutional schools (e.g., correctional, medical); (2) middle and high schools with fewer than 20 and 80 students, respectively; (3) geographically inaccessible schools; (4) classes with fewer than five students; special education classes, and English as a second language (ESL) classes. Students completed one of two alternately distributed (i.e., A.B.A) questionnaires (Form A or Form B). Both forms contained questions about TBI, smoking, alcohol, binge drinking, cannabis, and current marks in school. In addition to these full sample core items found in both forms A and B, Form A exclusively contained mental health, suicidality, and bullying questions (answered by a half sample of 4,685 students), whereas Form B exclusively contained the medically-treated injury item (answered by a half sample of 4,230 students). A complete description of the methods of the survey, including design, split-ballot questionnaires (forms A and B), discussion of the validity of self-reports, potential nonresponse bias, and limitations is available online [29].

### Traumatic brain injury (TBI)

Traumatic brain injury was assessed and defined as follows: *“We are interested in any head injuries that resulted in you being unconscious (knocked out) for at least 5 minutes, or you had to stay in the hospital for at least 1 night because of it. Did you have this type of head injury in your life?”* Responses included *(1) Yes, I′ve had a head injury like this in the last 12 months, (2) Yes, I′ve had a head injury like this in my life, but not in the last 12 months, or (3) No, I′ve never had a head injury like this in my life.”* For analysis, the first two responses were combined to represent lifetime prevalence and was binary coded (1,1,0). This definition of TBI is used in several classification systems including DSM-IV [30]–[34]. For analysis, responses 1 and 2 were combined to represent lifetime prevalence and were binary coded (1,1,0). This question, with an item response rate of 98%, is similar to those used in recent studies of self-reported TBI involving adults, although our study is the first general population survey involving adolescents [30], [35]. Although we have no external data to validate our TBI measure, we do have concurrent items that provide correlational evidence of validity. One such item concerns any injuries experienced in the past year that have been treated by a medical doctor. When correlating these items, a significantly positive relationship emerged (Cramer's V 0.21, P<.001). This correlation was both positive and significant, as expected. Moreover, we expect a modest correlation since the criterion includes injuries not restricted to TBI. To assess potential nonresponse bias, we compared high-participating classes (with 70% or more students in the class participating; n = 323 classes) to low-participating classes (less than 70% participating; n = 258 classes), and found no evidence of nonresponse bias (19.6 vs. 19.8, t_579_ = −0.189, P = 0.850).

### Past year cigarette smoking

The prevalence of past year cigarette smoking was assessed by asking if the student smoked at least one cigarette daily or smoked occasionally during the past 12 months [29]. Students who smoked a few puffs or less than one cigarette in the past 12 months were not classified as smokers (binary coded as 1-smokers, 0-non smokers).

### Past year daily smoking

Smoking at least one whole cigarette daily during the past 12 months was defined as daily smoking and was binary coded (1-daily smoker, 0-non daily smoker) [29].

### Past year alcohol use

Students were asked if they consumed any alcohol during the past 12 months [29]. Use includes consumption on special occasions, but excluding sips. Variable was binary coded (1-past year drinking, 0-no drinking in the past year).

### Recent binge drinking/heavy episodic drinking

Students were asked if in the past 4 weeks they engaged in drinking 5 or more drinks on the same occasion (binary coded as 1, 0-no engagement in drinking, 5+ drinks in one seating) [29].

### Past year cannabis use

Students were asked if they used cannabis at least once during the past 12 months (binary coded as 1) [29]. Cases that responded “don't know what [the drug] is” were classified nonusers (binary coded as 0) and assigned to the denominator.

### Current marks in school, on average

Students were asked, on average, what marks do they usually get in school [29]. Categories 1 (mostly A+: 90%–100%), 2 (mostly As or A−: 80%–89%), 3 (mostly Bs: 70%–79%) and 4 (mostly Cs: 60%–69%) were binary coded as 1 (60% or higher), and categories 5 (Mostly Ds: 50%–59%) and 6 (Mostly Fs: below 50%) were binary coded as 0 (below 60%).

### Elevated psychological distress

The 12-item General Health Questionnaire (GHQ12) was used to measure current elevated psychological distress collected in Form A [36]–[37]. The GHQ12 identifies depressed mood, anxiety, and social dysfunction. A cut score of three or more on the binary-scored GHQ12 is considered the validated threshold for identifying adolescents experiencing elevated psychological distress. Cronbach's reliability coefficient (α) for these 12 items in this sample is 0.89. The GHQ12 has been shown to be a valid screener among adolescents [36]–[37].

### Past 12 months suicide ideation

The suicide ideation question asked: “In the last 12 months, did you ever seriously consider attempting suicide?” Response options were yes or no. This question is from the Centre for Disease Control's Youth Risk Behaviour Survey (YRBS) and has demonstrated good reliability and validity among students [36], [38].

### Past 12 months bullying

Items measuring at school and cyber bullying assessed their past 12 months' occurrence, and were adapted from the World Health Organization's Health Behaviour of School-aged Children (HBSC) study [2], [36]. Bullying was defined as repeatedly being teased by one or more people, being hurt or upset, or being left out of things on purpose [36]. Students were asked if they were bullied at school since September. Response options included (1) was not bullied at school since September; (2) physical attacks (for example, beat you up, pushed or kicked you); (3) verbal attacks (for example, teased, threatened, spread rumours about you); (4) stole from you or damaged your things. Options 2 through 4 were combined to ensure sufficient cases. Cyber bullying was measured by asking, “In the last 12 months, how many times did other people bully or pick on you through the Internet?” Response options included (1) don't use the internet; (2) never; (3) once; (4) 2 or 3 times; (5) 4 or more times. Options 1 and 2, and 3 through 5 were combined to represent having been cyber bullied. Students were also queried if they bullied other students since September. Response options included (1) did not bully other students since September; (2) physical attacks (for example, beat up, pushed or kicked them); (3) verbal attacks (for example, teased, threatened, spread rumours about them); (4) stole from them or damaged their things. Options 2 through 4 were combined.

### Assessing drug use problems

Form A also included the 6-item CRAFFT screener that assesses drug use problems experienced by adolescents [39]. The six yes/no items pertain to problems experienced during the past year. Those endorsing two or more symptoms (binary coded as 1) identified adolescents as having a drug use problem.

### Past 12 months medically treated injuries

A measure of physical injuries that were treated by a medical professional was based on a question that asked students to report the number of times in the last 12 months they were “hurt or injured, and had to be treated by a doctor or nurse?” [29]. Responses ranged from 1 (was not treated) to 5 (4 or more times). A dichotomous measure representing treated at least once (coded 1) versus not treated (coded 0) for an injury was used for analysis.

To enhance anonymity and privacy, self-completed questionnaires, (which averaged 30 minutes to complete), were administered during a single class period by field staff of the Institute for Social Research, York University between November 2010 and June 2011. Active (or explicit) parental consent/student assent procedures were used. The final sample comprised 9,288 middle and high schoolers from 181 schools and 573 classes. This represents a participation of 71% among the selected schools and 62% of students in the selected classes.

### Analyses

Because our respondents were distributed among differing questionnaire panels of respondents who were asked differing sets of questions, our analyses were carried out in three stages. The first set of analyses used the full sample of 7th through 12th graders [N = 9,288 (age range 11–20; mean  = 15.1; SD = 1.82)], whereas the second and third sets employed the two half samples *Form A and Form B* [(n = 4,816 and 4,472, respectively), with mean ages of 14.9 (range: 11–20; SD = 1.81) and 14.8 (range: 11–20; SD = 1.84)], respectively. To accommodate the complex sampling of OSDUHS, which includes stratification, clustering and unequal selection probabilities necessitating the use of sampling weights, all analyses employed design-based estimation that accommodates the complex survey data (i.e., non-simple random sample data) as implemented in the Complex Sample module in SPSS V20.0, which produces correct estimates of variances (via Taylor series linearization) and statistical tests [40]–[41]. The pairwise associations between lifetime TBI and demographic and other risk factors were examined using design-based chi-square analyses. Further, we modeled three sets of logistic regressions, based on the total sample, and on the two subsamples.

To assess the moderating effects of sex and age for each outcome, we employed design-based binary logit regression, modeling our primary predictor lifetime TBI status (yes/no), with sex and age categories as the main effects and separately testing TBI by sex and TBI by sex by age interactions. With listwise deletion of item missing responses, the estimation samples were reduced from 9,288 to 8,915 (males = 51.7%) for the total sample analyses, from 4,816 to 4,685 (males = 50.3%) for subsample A, and 4,472 to 4,230 (males = 53.1%) for the treated injury question in subsample B.

## Results

Table 1 shows lifetime TBI cross-tabulated with our outcomes stratified by sex. As evidenced from the non-overlapping confidence intervals, the estimated prevalence of acquiring a lifetime TBI was greater for males than females (23.1% versus 17.1%), although not by an appreciable magnitude. Of the 13 pairwise cross-tabulations with significant TBI associations, nine were significant for both males and females, with four being significant only for females. In each instance the outcome were highest among adolescents who had sustained a TBI compared with those who had not.

**Table 1:** Lifetime prevalence of TBI for females and males by demographics and TBI associated factors, Ontario, Canada 7th–12th graders, 2011.

	Females			Males		
	n	Lifetime^a^ % 95 CI	Never % 95 CI	n	Lifetime^a^ % 95 CI	Never % 95 CI
***Total ^b^***	*4808*	*17.1 (14.7, 19.8)*	*82.9 (80.2,85.3)*	*4107*	*23.1 (20.5, 25.8)*	*76.9 (74.2,79.2)*
**Control variables**						
*Age*		*ns*		*ns*		
11–13	1276	15.7 (13.4, 18.3)	84.3 (81.7, 86.6)	1118	23.0 (19.7, 26.5)	77.0 (73.5, 80.3)
14–16	2444	17.3 (13.6, 21.8)	82.7 (78.2, 86.4)	2068	23.2 (20.1, 26.6)	76.8 (73.4, 79.9)
17–20	1088	18.1 (14.5, 22.4)	81.9 (77.6, 85.5)	921	22.9 (18.1, 28.6)	77.1 (71.4, 81.9)
**Associates of TBI**						
*Smoked cigarettes^b,c^*	***			*ns*		
Yes	373	16.1 (11.1,22.8)	6.4 (5.2,7.9)	385	12.6 (8.4,18.4)	8.4 (6.9,10.2)
No	4424	83.9 (77.2,88.9)	93.6 (92.1,94.8)	3712	87.4 (81.6,91.6)	91.6 (89.8,93.1)
*Smoked>1 cigarettes daily^b,c^*	***			*		
Yes	138	6.5 (3.9,10.5)	2.2 (1.5,3.2)	168	7.5 (4.7,11.9)	3.9 (2.9,5.1)
No	4659	93.5 (89.5,96.1)	97.8 (96.8,98.5)	3929	92.5 (88.1,95.3)	96.1 (94.9,97.1)
*Drank alcohol^b,c,d^*	***			***		
Yes	2533	67.1 (63.0,71.8)	53.1 (49.3,56.9)	2150	65.1 (60.0,70.0)	51.5 (48.6,54.4)
No	2275	32.9 (28.2,38.0)	46.9 (43.1,50.7)	1957	34.8 (30.0,40.0)	48.5 (45.6,51.4)
*Binge drinking^b,e^*	*			***		
Yes	936	30.2 (23.6,37.6)	20.0 (17.4,23.0)	906	31.3 (27.4, 35.5)	20.1 (17.4, 23.1)
No	3851	69.8 (62.4, 76.4)	80.0 (77.0,82.6)	3181	68.7 (64.5, 72.6)	79.9 (76.9, 82.6)
*Used cannabis^b,c^*	**			***		
Yes	947	30.4 (22.9,39.0)	19.1 (17.4,20.9)	955	33.3 (28.6, 38.5)	20.1 (17.4,23.1)
No	3861	69.6 (61.0,77.1)	80.9 (79.1,82.6)	3152	66.7 (61.5,71.4)	79.9 (76.9,82.6)
*Current marks in school, on average^b^ <60%*	**			***		
Yes	65	4.4 (2.1, 9.0)	1.2 (0.7,2.1)	77	4.8 (2.8, 7.9)	1.4 (0.9,2.1)
No	4743	95.6 (91, 97.9)	98.8 (97.9, 99.3)	4030	95.2 (92.1, 97.2)	98.6 (97.9,99.1)
***Subsample A*** *^f^*	*2541*	*16.6 (13.5, 20.3)*	*83.4 (79.7,86.7)*	*2144*	*22.2 (18.9, 25.9)*	*77.8 (74.1,81.1)*
*Elevated psychological distress^f^*	**			*ns*		
Yes	1498	53.4 (44.8,61.7)	41.5 (38.6,44.5)	499	29.3 (23.8, 35.4)	22.8 (20.2, 25.7)
No	1039	46.6 (38.3,55.1)	58.5 (55.5,61.4)	1638	70.7 (64.6, 76.2)	77.2 (74.3, 79,8)
*Contemplated suicide^c,f^*	***			*ns.*		
Yes	312	25.0 (19.2,31.8)	11.5 (9.7,13.6)	163	8.2 (5.0,13.3)	6.8 (5.3,8.7)
No	2208	75.0 (68.2,80.8)	88.5 (86.4,90.3)	1961	91.8 (96.7,95.0)	93.2 (91.3, 94.7)
*Bullied someone at school^f,g^*	**			*		
Yes	463	36.9 (23.1,53.1)	19.3 (15.7,23.6)	419	26.2 (19.0,35.0)	16.7 (13.9,19.9)
No	2050	63.1 (46.8,76.9)	80.7 (76.4,84.3)	1683	73.8 (65.0,81.0)	83.3 (80.1, 86.1)
*Been bullied at school^f,g^ (victim)*	**			*ns*		
Yes	775	46.9 (34.5,59.8)	28.3 (25.2,31.6)	530	29.2 (22.7,36.7)	24.3 (21.2,27.7)
No	1746	53.1 (40.2,65.5)	71.7 (68.4,74.8)	1584	70.8 (63.3,77.3)	75.7 (72.3, 78.8)
*Been bullied through the internet^c,f^*	**			**		
Yes	661	43.0 (30.3, 56.7)	24.8 (22.5,27.2)	316	20.9 (16.3,26.5)	13.1 (10.8,15.8)
No	1860	57.0 (43.3,69.7)	75.2 (72.8,77.5)	1796	79.1 (73.5, 83.7)	86.9 (84.2, 89.2)
*Drug use problems (CRAFFT)*	***			**		
Yes	296	20.5 (15.6,26.5)	10.0 (8.4,11.9)	315	18.4 (13.9,23.9)	12.1(9.4,15.3)
No	2242	79.5 (73.5,84.4)	90.0 (88.1,91.6)	1823	81.6 (76.1,86.1)	87.9 (84.7,90.6)
***Subsample B ^h^***	2249	17.7 (15.2, 20.5)	82.3 (79.5,84.8)	1952	23.9 (20.6, 27.5)	76.1 (72.5,79.4)
*Treated for physical injuries^c,h^*	***			***		
Yes	860	55.7 (49.6,61.7)	35.4 (30.9,40.2)	890	62.8 (56.8, 68.4)	38.4 (34.1, 42.0)
No	1389	44.3 (38.3,50.4)	64.6 (59.8,69.1)	1062	37.2 (31.6,43.2)	61.7 (58.0,65.3)

Note: ^a^ Percentages are weighted; ^b^ Totals are based on N = 8915, forms A and B of the survey; ^c^ Past 12 months; ^d^ Excluding sips; ^e^ Past four weeks; ^f^ Based on subsample A, n = 4685; ^g^ Since September; ^h^ Based on subsample B, n = 4230.

*ns* statistically not-significant, *p*>.05; * Rao-Scott adjusted Chi-square statistically significant, *p*<.05; ** Rao-Scott adjusted Chi-square statistically significant, *p*<.01; *** Rao-Scott adjusted Chi-square statistically significant, p<.001.

Table 2 further extends our sex-stratified analysis by regressing each of the 13 outcomes on TBI status, while holding constant the respondent's age category, which was modeled but not displayed. The results reveal several findings. First, for TBI the number of outcomes associated with lifetime TBI was greater for females than males. Indeed, for females, 13 of 13 outcomes were significantly associated with sustaining a lifetime TBI, whereas for males, only 9 of 13 TBI differences were statistically significant. The four outcomes showing TBI differences for females (but not males) include cigarette smoking, elevated distress, suicide ideation and being bullied. Although the presence of TBI was significant in predicting 9 of 13 outcomes for both males and females, four outcomes showed significant TBI differences for females only, whereas none of the TBI differences were discernible for males only. Second, it should also be noted that in all instances the observed effect sizes between covariates and TBI were higher for females than males. For females, TBI odds ratios range from 1.6 to 3.72 (median = 2.44, mean = 2.44) versus 1.3 to 3.52 (median = 2.18, mean = 2.0) for males. Among females, the top-ranked TBI odds ratios were for current marks in school below 60% (OR = 3.72), daily smoking (OR = 3.09) and past year smoking (OR = 2.83), and among males they were for current marks in school below 60% (OR = 3.52), medically treated bodily injuries (OR = 2.72) and cannabis use (OR = 2.19). Finally, the TBI difference is robust. For 9 of 13 outcomes, adjusting for the respondent's age did not appreciably alter the TBI univariate associations.

**Table 2:** Bivariate logistic regression of behavioural, substance use, mental health and academic performance by lifetime TBI, Ontario 7th–12th graders, 2011 (N = 8915).

	Females		Males	
	OR^a^ (95% CI)	*OR^b^ (95% CI)*	OR^a^ (95% CI)	*OR^b^ (95% CI)*
	***Smoked cigarettes*** *^c,d^*			
Never	1.00 (Reference)	*1.00 (Reference)*	1.00 (Reference)	*1.00 (Reference)*
Lifetime TBI	2.80 (1.95, 4.01) ^j^	*2.83 (1.94, 4.11)^j^*	1.57 (.92, 2.67)	*1.61 (.91, 2.85)*
	***Smoked>1 cigarettes daily*** *^c,d^*			
Never	1.00 (Reference)	*1.00 (Reference)*	1.00 (Reference)	*1.00 (Reference)*
Lifetime TBI	3.13 (1.99, 4.91)^ j^	*3.09 (1.97, 4.83)^j^*	2.01 (1.06, 3.82)^l^	*2.07 (1.07, 4.01) ^l^*
	***Drank alcohol*** *^c,d,e^*			
Never	1.00 (Reference)	*1.00 (Reference)*	1.00 (Reference)	*1.00 (Reference)*
Lifetime TBI	1.80 (1.44, 2.42) ^j^	*1.97 (1.51, 2.56) ^j^*	1.76 (1.37, 2.26) ^j^	*1.92 (1.45, 2.56) ^j^*
	***Binge drinking*** *^c,f^*			
Never	1.00 (Reference)	*1.00 (Reference)*	1.00 (Reference)	*1.00 (Reference)*
Lifetime TBI	1.72 (1.09, 2.72) ^l^	*1.77 (1.05, 3.00) ^l^*	1.82 (1.36, 2.42) ^j^	*2.00 (1.49, 2.69) ^j^*
	***Used cannabis*** *^c,d^*			
Never	1.00 (Reference)	*1.00 (Reference)*	1.00 (Reference)	*1.00 (Reference)*
Lifetime TBI	1.85 (1.24, 2.77)^k^	*1.92 (1.22, 3.03) ^k^*	1.99 (1.43, 2.77) ^j^	*2.19 (1.59, 3.04) ^j^*
	***Current marks in school, on average*** *^c^* ***<60%***			
Never	1.00 (Reference)	*1.00 (Reference)*	1.00 (Reference)	*1.00 (Reference)*
Lifetime TBI	3.71 (1.65, 8.36) ^k^	*3.72 (1.68, 8.27) ^k^*	3.52 (2.00, 6.20) ^j^	*3.52 (2.00, 6.19) ^j^*
	***Elevated psychological distress*** *^g^*			
Never	1.00 (Reference)	*1.00 (Reference)*	1.00 (Reference)	*1.00 (Reference)*
Lifetime TBI	1.61 (1.13, 2.29) ^k^	*1.62 (1.15, 2.30) ^k^*	1.40 (.99, 1.97)	*1.40 (.95, 2.06)*
	***Contemplated suicide*** *^d,g^*			
Never	1.00 (Reference)	*1.00 (Reference)*	1.00 (Reference)	*1.00 (Reference)*
Lifetime TBI	2.56 (1.68, 3.90) ^j^	*2.56 (1.70, 3.83) ^j^*	1.23 (.66, 2.31)	*1.19 (.60, 2.33)*
	***Bullied someone at school*** *^g,h^*			
Never	1.00 (Reference)	*1.00 (Reference)*	1.00 (Reference)	*1.00 (Reference)*
Lifetime TBI	2.44 (1.46, 4.10) ^j^	*2.44 (1.46, 4.08) ^j^*	1.77 (1.05, 3.00) ^l^	*1.77 (1.04, 3.00) ^l^*
	***Been bullied at school*** *^g,h^* ***(victim)***			
Never	1.00 (Reference)	*1.00 (Reference)*	1.00 (Reference)	*1.00 (Reference)*
Lifetime TBI	2.25 (1.29, 3.92) ^l^	*2.25 (1.29, 3.94) ^k^*	1.28 (.86, 1.91)	*1.29 (.86, 1.92)*
	***Been bullied through the internet*** *^c,h^*			
Never	1.00 (Reference)	*1.00 (Reference)*	1.00 (Reference)	*1.00 (Reference)*
Lifetime TBI	2.30 (1.33, 3.97) ^k^	*2.30 (1.33, 3.97) ^k^*	1.75 (1.15, 2.67) ^k^	*1.75 (1.15, 2.65) ^k^*
	***Drug use problems (CRAFFT)*** *^g^* ***(2+/6)***			
Never	1.00 (Reference)	*1.00 (Reference)*	1.00 (Reference)	*1.00 (Reference)*
Lifetime TBI	2.33 (1.73, 3.14) ^j^	*2.39 (1.69, 3.38) ^j^*	1.64 (1.13, 2.39) ^k^	*1.67 (1.10, 2.53) ^k^*
	***Treated for physical injuries*** *^i^*			
Never	1.00 (Reference)	*1.00 (Reference)*	1.00 (Reference)	*1.00 (Reference)*
Lifetime TBI	2.30 (1.67, 3.15) ^j^	*2.29 (1.66, 3.16) ^k^*	2.71 (1.96,3.76) ^j^	*2.72 (1.97, 3.75) ^j^*

Note: ^a^ Unadjusted odds-ratios (OR); ^b^ Adjusted for sex and age Odds-Ratios (OR); ^c^ Based on Surveys A and B, N = 8915; ^d^ Past 12 months; ^e^ Excluding sips; ^f^ Past 4 weeks; ^g^ Based on Form A of the survey, n = 4685; ^h^ Since September; ^i^ Totals are based on n = 4230 form B of the survey; *^j^* *** p<.001, 2 tails test; ^k^ ** p<.01, 2 tails test; ^l^ * p<.05, 2 tails test.

Three additional findings are of equal note. First we find that a cluster of four outcomes – smoking, elevated distress, suicide ideation, and being bullied – show a similar pattern of co-occurrence – statistically significant odds among females who sustained a TBI, but not among males. Additionally, for both males and females receiving poor current marks in school was strongly associated with a history of TBI; the ORs for history of TBI were greatest for students reporting current marks in school below 60% (ORs = 3.52 and 3.72, respectively). Finally, the odds of cannabis use were double for males sustaining a TBI (OR = 2.19) but also for females with TBI (OR = 1.92) versus males and females who did not sustain a TBI, respectively.

**Figure 1:**
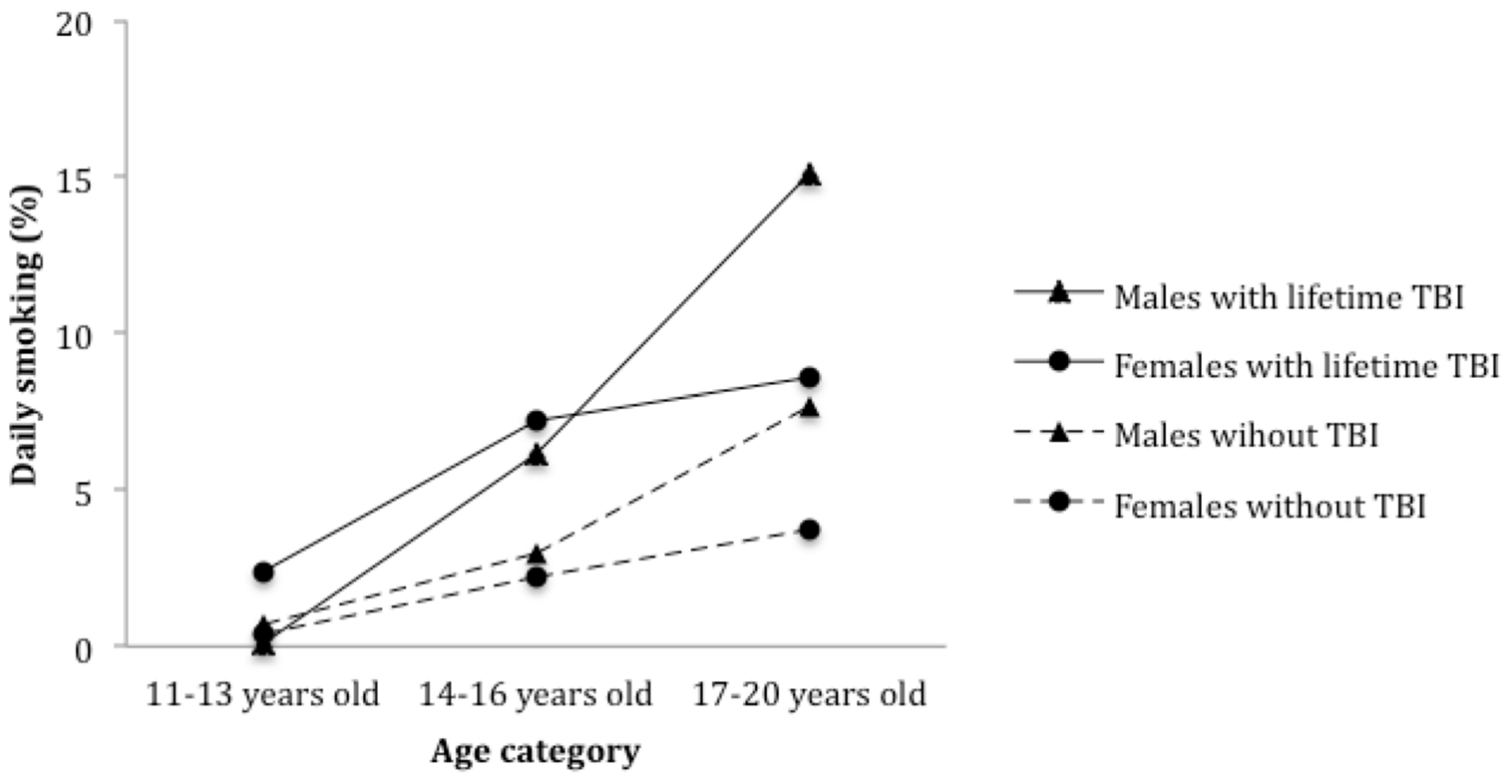
Past year daily smoking among adolescents with a history of TBI. Totals are based on N = 8915, forms A and B of the survey.

**Figure 2:**
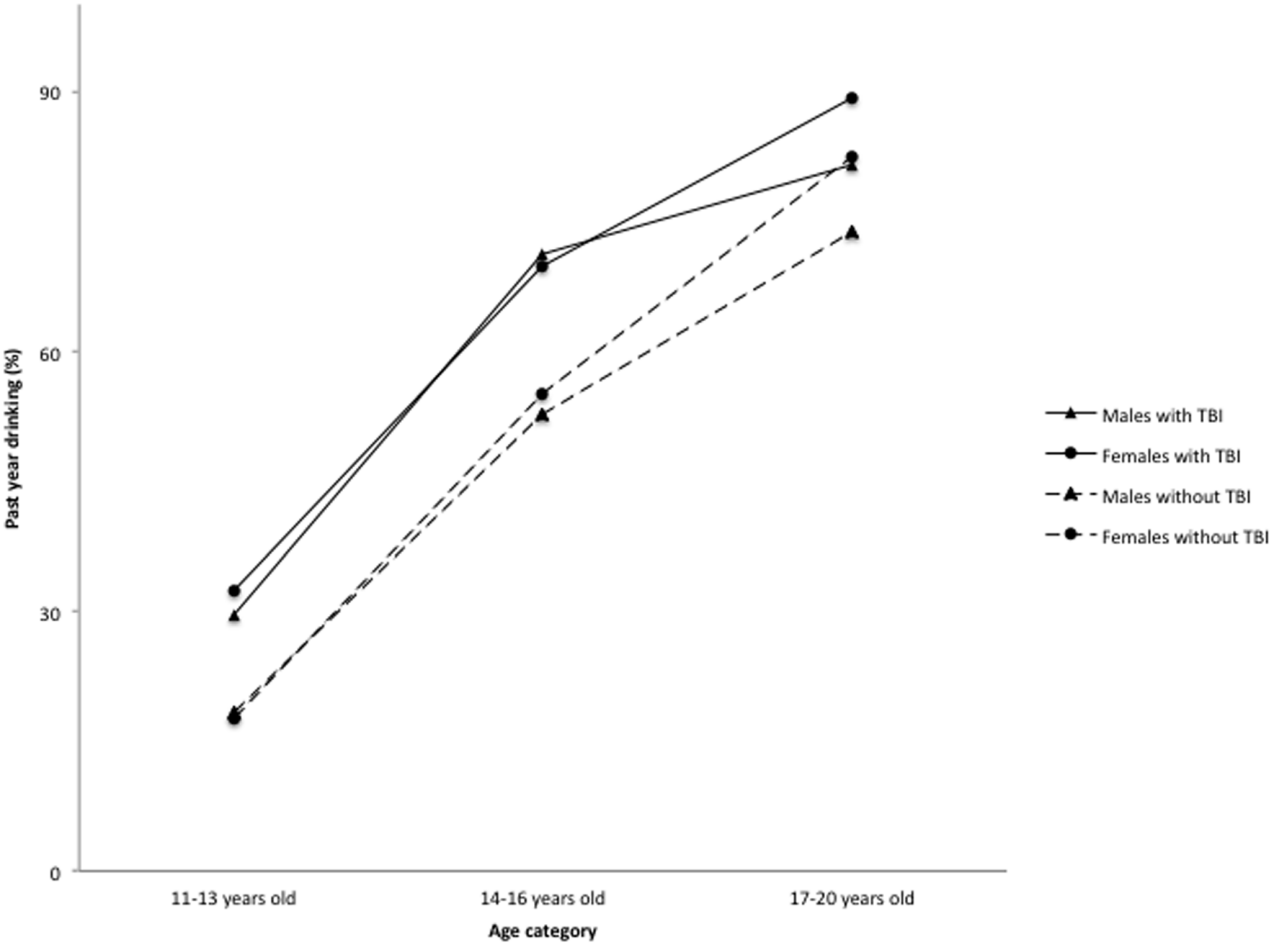
Past year drinking among adolescents with a history of TBI. Totals are based on N = 8915, forms A and B of the survey.

**Figure 3:**
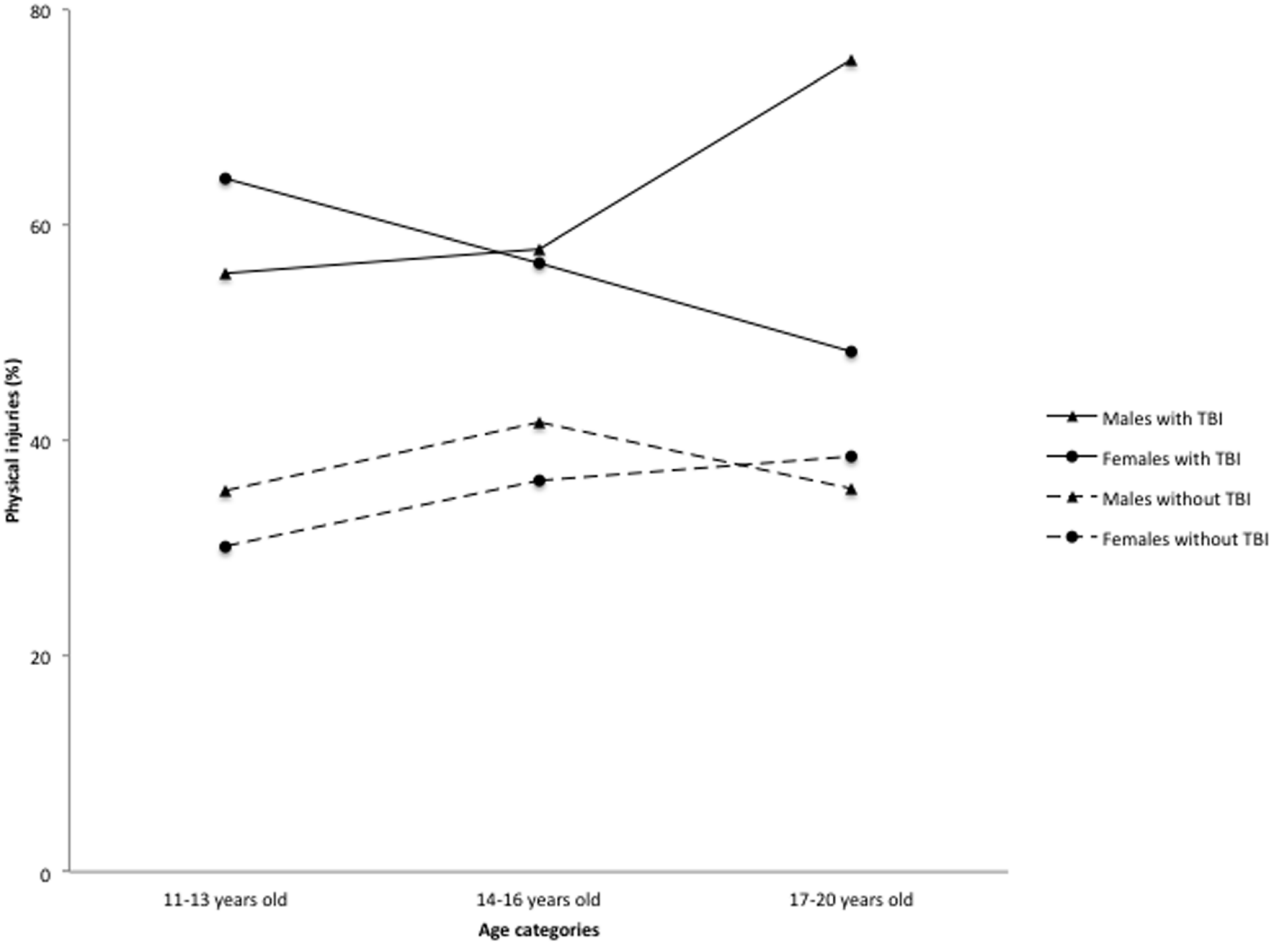
Past year physical injuries among adolescents with a history of TBI. Based on subsample B, n = 4230.

Table 3 depicts fitting the 2- and 3-way sex- (and age-) related interactions for the 13 outcomes. The distinguishing feature of the 3-way interaction involves the older 17 to 20 year olds. Although the prevalence of the 3 outcomes (daily smoking, alcohol use, and treated injuries), does not vary greatly between males and females with TBI for either the 11 to 13 year olds or the 14 to 16 year olds, moving from mid adolescence (14 to 16 years) to late adolescence (17 to 20 years) increases the sex difference in each of the 3 outcomes (Figures 1–3). Specifically, for females with TBI, daily cigarette use rose from 7.2% of 14 to 16 years olds to 8.6% of 17 to 20 years olds, whereas for males this increase rose more than 2 fold from 6.2% to 15.1% (Fig. 1). Similarly, among females with TBI, treated injuries declined from 56.5% to 48.2% for 14 to 16 year olds and 17 to 20 year olds, respectively, whereas among males injuries increased from 57.7% to 75.4% (Fig. 3). Finally, in the case of the TBI by sex by age interaction for alcohol use (Fig. 2), the transition from 14 to 16 years to 17 to 20 years widened the sex difference in alcohol use, but with females surpassing males (from 71.2% to 81.6% for males and from 69.8% to 89.3% for females). For the TBI by sex interaction, we found that the female TBI OR (2.83, 95% CI: 1.94, 4.11) is statistically significant for cigarette smoking in the past 12 months, but the male TBI OR is not (1.61, 95% CI:.91, 2.85).

**Table 3:** Logistic Regression modeling with TBI, sex and Age and two interaction terms.

	Model 1		Model 2	
	ORs (95% CI)	Wald Statistic	ORs (95% CI)	Wald Statistics
	***Smoked cigarettes*** a **^,^** ^***b***^			
*TBI*		*F* (1, 166) = 13.45^h^		*F* (1, 166) = 13.16^h^
Never	1.00 (Reference)		1.00 (Reference)	
Lifetime	2.81 (1.96, 4.02)		1.94 (1.01, 3.73)	
*Sex*		*F* (1, 166) = 0.08		*F* (1, 166) = 0.08
Male	1.00 (Reference)		1.00 (Reference)	
Female	1.40 (0.97, 2.03)		0.93 (0.56, 1.55)	
*Age*		*F* (2,165) = 29.98^h^		*F* (2, 165) = 18.65^h^
11–13	1.00 (Reference)		1.00 (Reference)	
14–16	4.26 (2.37, 7.65)		3.56 (1.19, 10.59)	
17–20	9.53 (5.14, 17.68)		4.57 (1.24, 16.85)	
TBI x Sex		*F* (1, 166) = 5.21^i^		*F* (1, 166) = 1.92
TBI x Sex x Age		–		*F* (6, 161) = 0.61
	**Smoked>1 cigarettes daily** a **^,b^**			
TBI		*F* (1, 166) = 14.75^h^		*F* (1, 166) = 5.22^i^
Never	1.00 (Reference)		1.00 (Reference)	
Lifetime	3.07 (1.98, 4.75)		2.44 (1.27, 4.70)	
Sex		*F* (1, 166) = 2.38		*F* (1, 166) = 0.24
Male	1.00 (Reference)		1.00 (Reference)	
Female	0.86 (0.49, 1.52)		0.53 (0.29, 0.96)	
Age		*F* (2, 165) = 21.98^h^		*F* (2, 165) = 29.88^h^
11–13	1.00 (Reference)		1.00 (Reference)	
14–16	5.77 (2.40, 13.84)		3.68 (0.90, 15.11)	
17–20	12.55 (5.50, 28.60)		4.46 (1.18, 16.78)	
TBI x Sex		*F* (1, 166) = 1.89		*F* (1, 166) = 10.15^i^
TBI x Sex x Age		–		*F* (6, 161) = 2.65^i^
	**Drank alcohol** a ^,b,c^			
TBI		*F* (1, 166) = 42.24^h^		*F* (1, 166) = 35.70^h^
Never	1.00 (Reference)		1.00 (Reference)	
Lifetime	1.92 (1.51, 2.43)		1.77 (0.89, 3.55)	
Sex		*F* (1,166) = 3.52		*F* (1, 166) = 5.15*
Male	1.00 (Reference)		1.00 (Reference)	
Female	1.13 (0.85, 1.49)		1.88 (0.90, 3.94)	
Age		*F* (2, 165) = 176.61^h^		*F* (2, 165) = 150.21^h^
11–13	1.00 (Reference)		1.00 (Reference)	
14–16	5.39 (4.28, 6.80)		4.81 (2.85, 8.12)	
17–20	15.62 (11.66, 20.91)		17.45 (7.45, 40.85)	
TBI x Sex		*F* (1, 166) = 0.08		*F* (1, 166) = 0.064
TBI x Sex x Age		–		*F* (6, 161) = 3.58^i^
	**Binge drinking** a **^,d^**			
TBI		*F* (1, 166) = 38.01^h^		*F* (1, 166) = 27.64^h^
Never	1.00 (Reference)		1.00 (Reference)	
Lifetime	1.76 (1.06, 2.91)		1.44 (0.58, 3.56)	
Sex		*F* (1, 166) = 0.06		*F* (1, 166) = 0.80
Male	1.00 (Reference)		1.00 (Reference)	
Female	0.96 (0.60, 1.55)		0.86 (0.26, 2.80)	
Age		*F* (2, 165) = 167.24^h^		*F* (2, 165) = 109.20^h^
11–13	1.00 (Reference)		1.00 (Reference)	
14–16	13.22 (8.53, 20.49)		8.14 (3.11, 21.28)	
17–20	34.01 (21.98, 52.62)		15.62 (4.45, 54.74)	
TBI x Sex		*F* (1, 166) = 0.08		*F* (1, 166) = 0.09
TBI x Sex x Age		–		*F* (6, 161) = 0.68
	**Used cannabis** a **^,c^**			
TBI		*F* (1, 166) = 44.39^h^		*F* (1, 666) = 43.63h
Never	1.00 (Reference)		1.00 (Reference)	
Lifetime	1.89 (1.22, 2.92)		2.09 (1.23, 3.55)	
Sex		*F* (1, 166) = 0.29		*F* (1, 166) = 1.77
Male	1.00 (Reference)		1.00 (Reference	
Female	0.87 (0.51, 1.51)		0.92 (0.44, 1.92)	
Age		*F* (2, 165) = 114.77^h^		*F* (2, 165) = 90.94^h^
11–13	1.00 (Reference)		1.00 (Reference)	
14–16	7.36 (4.87, 11.11)		7.13 (2.60, 19.55)	
17–20	17.80 (11.81, 26.83)		18.67 (6.83, 51.04)	
TBI x Sex		*F* (1, 166) = 0.16		*F* (1, 166) = 0.35
TBI x Sex x Age		–		*F* (6, 161) = 1.28
	**Current marks in school, on average** a **<60%**			
TBI		*F* (1, 166) = 19.99^h^		*F* (1, 666) = 18.58^h^
Never	1.00 (Reference)		1.00 (Reference)	
Lifetime	3.70 (1.67, 8.22)		4.14 (.77, 22.35)	
Sex		*F* (1, 166) = 0.16		*F* (1, 166) = 0.13
Male	1.00 (Reference)		1.00 (Reference	
Female	0.93 (0.48, 1.78)		0.78 (0.13, 4.56)	
Age		*F* (2, 166) = 0.02		*F* (2, 165) = 0.22
11–13	1.00 (Reference)		1.00 (Reference)	
14–16	1.06 (0.47, 2.30)		1.48 (0.39, 5.63)	
17–20	1.25 (0.52, 2.98)		1.45 (0.23, 9.30)	
TBI x Sex		*F* (1, 165) = 0.26		*F* (1, 166) = 0.04
TBI x Sex x Age		–		*F* (6, 161) = 0.42
	**Elevated psychological distress** ^e^			
TBI		*F* (1, 166) = 11.34^i^		*F* (1, 666) = 12.32^h^
Never	1.00 (Reference)		1.00 (Reference)	
Lifetime	1.63 (1.14, 2.32)		1.61 (0.92, 2.83)	
Sex		*F* (1, 166) = 71.01^h^		*F* (1, 166) = 48.33^h^
Male	1.00 (Reference)		1.00 (Reference)	
Female	2.92 (1.82, 4.66)		2.32 (0.80, 6.74)	
Age		*F* (2, 165) = 35.00^h^		*F* (2, 165) = 16.71^h^
11–13	1.00 (Reference)		1.00 (Reference)	
14–16	1.88 (1.45, 2.43)		2.07 (0.94, 4.57)	
17–20	2.94 (2.28, 3.80)		2.73 (1.14, 6.58)	
TBI x Sex		*F* (1, 166) = 0.25		*F* (1, 166) = 0.01
TBI x Sex x Age		–		*F* (6, 161) = 0.99
	**Contemplated suicide** ^b,e^			
TBI		*F* (1, 166) = 13.57^h^		*F* (1, 666) = 9.20^i^
Never	1.00 (Reference)		1.00 (Reference)	
Lifetime	2.61 (1.75, 3.90)		2.64 (1.11, 6.29)	
Sex		*F* (1, 166) = 34.31^h^		*F* (1, 166) = 21.00^h^
Male	1.00 (Reference)		1.00 (Reference	
Female	3.83 (1.93, 7.62)		3.03 (.75, 12.34)	
Age		*F* (2, 165) = 4.05^i^		*F* (2, 165) = 2.81
11–13	1.00 (Reference)		1.00 (Reference)	
14–16	1.06 (0.00, 2.86)		3.45 (1.55, 7.66)	
17–20	1.77 (1.77, 1.77)		3.12 (1.09, 8.89)	
TBI x Sex		*F* (1, 166) = 2.79		*F* (1, 166) = 1.73
TBI x Sex x Age		–		*F* (6, 161) = 1.36
	**Bullied someone at school** ^e,f^			
TBI		*F* (1, 166) = 10.04^i^		*F* (1, 666) = 10.49^i^
Never	1.00 (Reference)		1.00 (Reference)	
Lifetime	2.45 (1.47, 4.07)		2.04 (0.81, 5.14)	
Sex		*F* (1, 166) = 4.06^i^		*F* (1, 166) = 2.67
Male	1.00 (Reference)		1.00 (Reference)	
Female	1.66 (1.00, 2.74)		2.65 (0.48, 14.73)	
Age		*F* (2, 165) = 2.13		*F* (2, 165) = 2.17
11–13	1.00 (Reference)		1.00 (Reference)	
14–16	1.39 (0.99, 1.96)		2.09 (0.83, 5.28)	
17–20	2.12 (0.81, 1.83)		1.45 (0.36, 5.88)	
TBI x Sex		*F* (1, 166) = 1.76		*F* (1, 166) = .86
TBI x Sex x Age		–		*F* (6, 161) = .83
	**Been bullied at school** ^e,f^ **(victim)**			
TBI		*F* (1, 166) = 9.80^i^		*F* (1, 166) = 9.79^i^
Never	1.00 (Reference)		1.00 (Reference)	
Lifetime	2.26 (1.29, 3.95)		2.20 (0.99, 4.91)	
Sex		*F* (1, 166) = 12.40^i^		*F* (1, 166) = 11.56^i^
Male	1.00 (Reference)		1.00 (Reference)	
Female	2.14 (1.21, 3.78)		4.53 (1.17, 17.50)	
Age		*F* (2, 165) = 2.48		*F* (2, 165) = 2.89
11–13	1.00 (Reference)		1.00 (Reference)	
14–16	0.94 (0.71, 1.24)		0.88 (0.36, 2.13)	
17–20	0.75 (0.54, 1.06)		0.88 (0.28, 2.71)	
TBI x Sex		*F* (1, 166) = 2.54		*F* (1, 166) = 2.30
TBI x Sex x Age		–		*F* (6, 161) = 1.25
	**Been bullied through the internet** a ^,f^			
TBI		*F* (1, 166) = 12.82^h^		*F* (1, 666) = 16.07^h^
Never	1.00 (Reference)		1.00 (Reference)	
Lifetime	2.29 (1.34, 3.94)		2.67 (1.25, 5.71)	
Sex		*F* (1, 166) = 29.99^h^		*F* (1, 166) = 28.29^i^
Male	1.00 (Reference)		1.0 (Reference	
Female	2.87 (1.62, 5.07)		1.94 (0.91, 4.13)	
Age		*F* (2, 165) = 0.51		*F* (2, 165) = 0.44
11–13	1.00 (reference)		1.00 (Reference)	
14–16	1.14 (0.85, 1.54)		1.18 (0.49, 2.81)	
17–19	1.19 (0.79, 1.81)		1.24 (0.39, 3.94)	
TBI x Sex		*F* (1, 166) = 0.81		*F* (1, 166) = 0.91
TBI x Sex x Age		–		*F* (6, 161) = 1.03
	**Drug use problems (CRAFFT)** ^e^ **(2+/6)**			
TBI		*F* (1, 166) = 29.23^h^		*F* (1, 666) = 11.02^i^
Never	1.00 (Reference)		1.00 (Reference)	
Lifetime	2.39 (1.68, 3.42)		2.08 (1.13, 3.84)	
Sex		*F* (1, 166) = 0.01		*F* (1, 166) = 0.01
Male	1.00 (Reference)		1.0 (Reference	
Female	1.20 (0.81, 1.77)		0.85 (0.41, 1.75)	
Age		*F* (2, 165) = 29.77^h^		*F* (1, 166) = 0.24
11–13	1.00 (Reference)		1.00 (Reference)	
14–16	6.40 (3.77, 10.86)		16.49 (4.35, 62.54)	
17–20	11.02 (5.92, 20.49)		16.76 (3.77, 74.50)	
TBI x Sex		*F* (1, 166) = 1.65		*F* (1, 165) = 29.02^h^
TBI x Sex x Age		*–*		*F* (6, 161) = 1.40
	**Treated for physical injuries** ^g^			
TBI		*F* (1, 159) = 83.51^h^		*F* (1, 159) = 66.59^h^
Never	1.00 (Reference)		1.00 (Reference)	
Lifetime	2.28 (1.66, 3.14)		1.48 (0.67, 3.26)	
Sex		*F* (1, 159) = 4.15^i^		*F* (1, 160) = 2.96
Male	1.00 (Reference		1.00 (Reference)	
Female	0.74 (0.52, 1.06)		0.30 (.14,.68)	
Age		*F* (2, 165) = 2.27		*F* (2,165) = 0.37
11–13	1.00 (Reference)		1.00 (Reference)	
14–16	1.22 (1.01, 1.47)		0.72 (0.38, 1.36)	
17–20	1.22 (0.91, 1.65)		0.52 (0.19, 1.40)	
TBI x Sex		*F* (1, 159) = 0.47		*F* (1, 159) = 0.59
TBI x Sex x Age		*–*		*F* (23, 137) = 2.51^i^

a Based on Surveys A and B, N = 8915; ^b^ Past 12 months; ^c^ Excluding sips; ^d^ Past 4 weeks; ^e^ Based on Form A of the survey, n = 4685; ^f^ Since September; ^g^ Totals are based on n = 4230 form B of the survey; ^h^ *** p<.001, 2 tail test; ^i^ ** p<.01, 2 tail test; ^j^ * p<.05, 2 tails test.

## Discussion

One in five adolescents reported sustaining a lifetime TBI that resulted in a loss of consciousness for at least five minutes or an overnight hospitalization due to symptoms. Males reported more TBIs in their lifetime by six percentage points compared to females. The most commonly reported cause of TBI for our sample was team sports [8]. Our prevalence estimate of 20.2% is some 10 percentage points lower than the retrospective self-reported lifetime TBI estimates seen in some previous studies of older adolescents and young adults employing non-representative or clinical samples [12], [42]–[44]. This estimate is also about 10 percentage points lower than a birth cohort study from New Zealand who captured the hospitalized as well as non-hospitalized TBI events of 1265 individuals from the time they were born to age 25 [1]. The former difference may be due to two factors; our wider and younger age range (11 to 20 years old) that includes early and mid-adolescence, as well as our definition of TBI, which excluded forms of injury not involving loss of consciousness or at least one overnight hospital stay. Further, the OSDUHS exclusion of students in special education classes may have excluded groups with elevated TBI. The latter difference, may be due to cross-cultural differences as well as differences in the age sampling selection (5 years difference between our studies' highest age included). Possible cross-cultural differences between our samples are suggested by the mechanism of injury reported. In our sample, most injuries were due to team sports (63% for males and 46.9% for females), falls (24.7% for females, 5.1% for males) and bicycle accidents (8.1% for males, 1.8% for females) [8]. In the New Zealand sample, for individuals 0–15 years of age, most injuries were due to falls (66.96%) and being hit by an object (10.13%). For youth between 15–25, most injuries happened while playing rugby (21.2%), due to assaults (19.9%) or as a result of motor vehicle accidents (23.4%) [1]. However, it is important to note that mechanisms of injury in the New Zealand sample reflect lifetime injuries, whereas in our sample they only reflect injuries reported in the past 12 months.

Adolescent males and females who reported lifetime TBI also reported past year concurrent daily smoking, drinking, binge drinking, using cannabis, drug use problems, cyberbullying, bullying others, poor current marks in school, and being treated for physical injuries, compared to their peers who did not report a TBI. These results indicate that adolescents with TBI are vulnerable to a range of psychological and behavioural harms that co-occur with their history of a TBI. The association between the range of adverse correlates identified here and TBI suggests that the adverse correlates could represent a coping mechanism to deal with the effects of TBI, or that they may predispose adolescents to TBI or perhaps both [8], [17], [45]. For example, early substance abuse has been found to be a mediating factor for criminal involvement of children and youth with a history of TBI [44]. The potential for negative synergistic effects of combined TBI and mental health and substance problems, including impact on academic performance, social and vocational failure, suggests that this should be a priority area for further investigation [1], [6], [8], [17], [20].

Similarly, a cross-sectional study of 5000 adults from New Haven, Connecticut, found that 7.2% of the adults interviewed recalled a blow to the head, which caused confusion or unconsciousness and these individuals were twice as likely as non injured individuals to suffer from alcoholism, drug abuse, major depression and other psychiatric conditions, along with increased rates of panic attacks and suicide attempts [46]. Since these conditions may take time to develop, parents and children may forget a prior TBI that preceded their occurrence and not link them during medical appointments and reviews of the history leading to the emergence of these conditions, hence medical vigilance on this matter is warranted and recommended.

Female adolescents with lifetime TBI may be more vulnerable for the co-existence of adverse psychological and behavioural conditions than male adolescents with TBI. We found that adolescent females who reported a lifetime TBI were significantly more likely to report four more health-compromising outcomes than males even when holding constant the effect of age. These adverse associations were current elevated psychological distress, past year suicidal ideation, being bullied at school in the past year and past-year cigarette smoking. Further analyses describing the interactive effects among TBI, sex and age showed earlier risk for girls with lifetime TBI to also report daily cigarette smoking, compared with girls without TBI, an effect that was not evident for boys. In the total sample, smokers report smoking their first cigarette at an average age of 14 [29]. Our analysis of the TBI positive group shows that girls, but not boys, with TBI had an earlier onset of smoking compared with girls without TBI. Higher odds associated with past year drinking were evident among adolescent girls and boys with TBI among ages 11 through 13 as well as 14 through 16 compared with those who did not report any TBI. Among 7th graders, 13% had their first drink before the end of grade 6 [29]. We observe here that drinking at an early age is particularly prevalent among adolescents with TBI. The highest odds associated with physical injuries treated by a physician were observed in early adolescence (11 through 13) for girls with TBI and later adolescence (17 through 20) for boys with TBI compared with their counterparts without TBI. In 2011, 44.2% of males and 39.3% of females reported physical injuries that were treated by a physician [29]. We observed here that students with TBI were at higher risk for physical injuries that required treatment by a physician. This risk is particularly higher among young girls age 11 through 13 and older boys age 17 through 20.

Our results are concordant with those of a meta-analysis showing elevated vulnerability for post-TBI symptoms among adult women following mild forms of TBI in adult investigations [19]. Possible bio-social factors that may be responsible for gender differences in outcomes post TBI include premorbid differences, post TBI differences in treatments, hormonal differences, differences in cognitive abilities and psychosocial factors, or a combination of these factors [19]. Future studies should investigate whether these differences could be explained by neurochemical, neuroanatomical or developmental changes post-TBI between sexes. Further, the circumstances around the history of the TBI should be investigated and examined to see if childhood trauma such as a history of abuse or neglect may be associated with TBI and its adverse correlates (46,47). Higher risk for related comorbidities following TBI may also affect the long-term quality of life of these individuals and should be examined by future research [49]. Long-term outcome studies have shown an elevated rate of smoking and drinking among female TBI survivors in relation to the general population [51]. The strong associations among smoking, alcohol consumption or both and breast cancer in women are well known [49]. Data from adult studies, and now from adolescent data, show that women and girls with TBI report concurrent smoking, drinking and binge drinking conditions compared to women and girls without TBI [8], [16], [19], [30], [48]. It is possible that binge drinking rates for girls in this study may have been underestimated compared to other studies. The definition of binge drinking that has been used in the OSDUHS is the same for both males and females (5 or more drinks on an occasion). However, the National Institute on Alcohol Abuse and Alcoholism has suggested that a binge occasion for women be defined as 4 or more drinks in one setting for girls/women, and 5 or more drinks for boys/men [49]. A recent report by the CDCP, revealed that binge drinking (using the NIAAA operational definition of 4 or more drinks in one setting, for women) among young girls was around 20% (one in five) and for women around 24% (one in four) in the US [50]. Nonetheless, our study provides additional support for sex and gender based analyses in TBI research [22], [27], [51].

Further analyses describing the interactive effects between TBI, sex and age showed that late-adolescent males with a lifetime TBI report elevated daily smoking and injuries, whereas late-adolescent females with a lifetime TBI report elevated alcohol use. Female adolescents with TBI had daily cigarette smoking estimates that were 1.4 percentage points different between the upper age categories: 14 to 16 years olds and 17 to 20 years olds. For males, however, the increase was 8.9 percentage points higher between the 14 to 16 years old and 17 to 20 years old age categories. TBI differences among medically treated injuries were lower by 16.2% between the 11–13 year old age group and the 17–20 years old age group for females, but were higher by almost 20% for males. Lastly, the transition from 14 to 16 years to 17 to 20 years widened the sex difference with females reporting higher alcohol consumption than males (89.3% v 81.65). The clinical implications here include promoting vigilance and screening for potential substance use and physical injuries in adolescent patients with TBI. Primary physicians should also inquire about past TBIs among adolescents reporting these conditions during initial consultation. Efforts to prevent TBI during adolescence and intervene at an early stage may reduce injuries and comorbid problems in this age group. Predicting outcomes and providing care for adolescents with TBI remains problematic, in the absence of clinical data. To address this public health issue, a refocusing of research efforts on this population is justified to prevent the re-occurrence of TBI over time and to discern unique care requirements to facilitate best outcomes.

### Limitations

Our findings, of course, are bounded by limitations of the study, the most salient of which is our self-report data gathering, which could affect the estimation of TBI prevalence and its associations. Preliminary analyses, however, found no evidence of nonresponse bias in the reporting of TBI; reports of TBI did not differ between classes with response rates above 70% and those with lower rates. As well, our sample excluded groups of adolescents who might be at high risk for TBI (such as those institutionalized). Also our study did not assess TBI severity. Thus, if males and females have differences in TBI severity, we cannot know whether our findings are confounded. Finally, even though the cross-sectional nature of our data precludes causal inferences, our investigation of moderating effects should not be impaired. Future studies, however, may consider longitudinal and prospective approaches that can establish causality and may determine antecedents and consequences of injury by employing multiple quantitative (e.g., personality and neuropsychological assessments), qualitative and innovative technologies (e.g., MRI).

### Contributions

Despite such limitations, we believe this study has made important contributions to the field by demonstrating that sex differences in the associations between TBI and health measures may be altered by age, and may depend on the measure considered. This is the first investigation examining sex differences among adolescents with TBI and adverse psychological conditions to use a large and representative population sample. The results point to important opportunities for prevention such as early-stage medical interventions, as well as diagnosis and rehabilitation approaches that consider the relationship between TBI and its risk factors. We know both adolescent boys and girls with TBI are vulnerable to the co-occurrence of harmful psychological and behavioural conditions [8], [16], [17]. Given that most brain injuries among adolescents occur during sports a more ecological approach to injury prevention requiring involvement by government, schools and parents, may be needed [5]–[8]. This would encompass involvement by government committees, schools and parents [7]. Sports related head injuries, as well as many or most injuries are preventable. Our findings suggest that more research on the potential moderating effects of sex and age on adolescent TBI outcomes is needed.
